# Physical and Performance Characteristics of Elite Youth Male Basketball Players Characterized by Maturity Status

**DOI:** 10.3390/life16010040

**Published:** 2025-12-26

**Authors:** Denis Čaušević, Monica Delia Bîcă, Amila Hodžić, Alina Elena Albină, Blake Densley, Dan Iulian Alexe, Milan Zelenović, Marta Bichowska-Pawęska, Mirza Ibrahimović, Cătălin Vasile Savu

**Affiliations:** 1 Faculty of Sport and Physical Education, University of Sarajevo, 71000 Sarajevo, Bosnia and Herzegovina; denis.causevic@fasto.unsa.ba (D.Č.); amila.hodzic@fasto.unsa.ba (A.H.); mirza.ibrahimovic@fasto.unsa.ba (M.I.); 2 Faculty of Medical and Behavioral Sciences, “Constantin Brâncuși” University of Târgu Jiu, 210185 Târgu Jiu, Romania; 3 Department of Theory and Methodology of Motor Activities, University of Craiova, 200585 Craiova, Romania; alina.albina@edu.ucv.ro; 4 School of Kinesiology, College of Health Sciences, Boise State University, Boise, ID 83725-1742, USA; blakedensley@boisestate.edu; 5 Department of Physical and Occupational Therapy, “Vasile Alecsandri” University of Bacau, 600115 Bacău, Romania; 6 Faculty of Physical Education and Sport, University of East Sarajevo, 71123 Istočno Sarajevo, Bosnia and Herzegovina; milan.zelenovic@ffvis.ues.rs.ba; 7 Faculty of Physical Education, Gdansk University of Physical Education and Sport, 80-336 Gdansk, Poland; marta.bichowska@awf.gda.pl; 8 Department of Sport Games and Physical Education, “Dunărea de Jos” University of Galati, Str. Domnească, nr. 47, 800008 Galați, Romania; catalin.savu@ugal.ro

**Keywords:** peak height velocity (PHV), talent identification, team sports, anthropometry, youth sport

## Abstract

This study investigated the influence of biological maturity status on anthropometric, body composition, and physical performance characteristics in elite youth male basketball players. A total of 140 players (15.12 ± 0.78 years) competing in national elite programs were categorized as early, on-time, or late maturers according to years from peak height velocity (PHV). Each participant completed a standardized testing battery including anthropometric assessments, body composition analysis (InBody 720), countermovement jump (CMJ) with and without arm swing, drop jump from 40 cm (DJ40), linear sprints over 5–20 m, and agility tests (*t*-test and Lane Agility). Between-group differences were analyzed using one-way ANOVA and Bonferroni post hoc tests, while partial eta squared (ηp^2^) and magnitude-based inference (MBI) were applied to assess effect size and practical significance. Significant differences were observed across maturity groups (*p* < 0.05), with early maturers being taller, heavier, and more muscular than their on-time and late-maturing peers. Large effects were found for height (ηp^2^ = 0.667) and body mass (ηp^2^ = 0.455), and moderate-to-large effects for jump, sprint, and agility performance (ηp^2^ = 0.051–0.166). MBI results indicated that most differences between early and late maturers were “very likely” or “almost certain,” highlighting their practical relevance. These findings confirm that biological maturity substantially affects physical and performance profiles in adolescent basketball players and underscore the importance of maturity-informed approaches such as bio-banding and individualized training to ensure fair evaluation and equitable talent development in youth sport.

## 1. Introduction

Basketball is a multifactorial sport that demands the coordinated development of anthropometric, physiological, technical, and psychological qualities to achieve elite performance levels [1,2]. During adolescence, these characteristics are strongly influenced by the interaction between biological growth, maturation, and accumulated training experience [3,4]. This period of development is crucial for both athletic progression and talent identification, but it is also marked by large differences among individuals in their biological and functional capacities [3,4].

The identification and selection of young basketball players typically occur between the ages of 12 and 16—a phase marked by wide differences in the rate and timing of maturation among athletes of the same chronological age [5]. Such differences, which may span several years, lead to considerable variation in body size, muscle development, hormonal profile, and neuromuscular capacity [6]. Consequently, early-maturing players often display temporary advantages in height, strength, and speed compared with later-maturing peers. This can lead to the overrepresentation of early maturers in elite programs—not necessarily because of greater long-term potential, but due to short-term physical advantages linked to earlier biological development [3,7]. To account for these differences, biological maturity can be estimated using years from peak height velocity (PHV), which represents how many years an athlete is before or after their fastest period of growth in height, an important characteristic for basketball performance. PHV-based maturity assessment is preferred in applied youth sport settings because it is non-invasive and practical for large field-based samples. This method is more sensitive than chronological age for identifying the wide variation in biological maturity common among players aged 12 to 16 years and provides a practical way to classify athletes as early, on-time, or late maturers [7,8].

From a physiological perspective, maturation brings a series of morphological and neuromuscular adaptations, including increases in limb length, muscle cross-sectional area, and lean mass, along with hormonal changes—particularly elevated testosterone—that collectively enhance strength and power output [9,10]. These adaptations directly influence basketball-specific actions such as jumping, sprinting, and rapid changes in direction, all of which rely on lower-limb explosive strength, coordination, and reactive power. Understanding how biological maturity influences these attributes provides valuable insight for coaches seeking to interpret player performance and design developmentally appropriate training interventions [11,12].

Although early maturers often appear dominant during adolescence, these physical advantages do not necessarily predict long-term success. Studies in team sports have shown that talent selection processes often favor early developers, leading to the premature exclusion of late maturers who may possess equal or greater potential once they reach full maturity [7,13,14]. This selection bias undermines the principles of long-term athlete development and narrows the potential talent pool at the senior level. To address this problem, maturity-informed strategies such as bio-banding and maturity-offset classification have been introduced to align training and competition with an athlete’s biological rather than chronological age [4]. These methods aim to create fairer environments and to support equal opportunities for development across all maturity levels.

Despite increasing awareness of the role of maturation in youth sport, relatively few studies have examined how biological maturity affects the anthropometric and physical performance profiles of elite youth basketball players. Most existing research has involved small or region-specific samples, limited testing protocols, or lacked subgrouping by maturity status, reducing the applicability of findings to broader elite populations [15,16,17]. Consequently, basketball still lacks maturity-specific reference data showing how body composition and performance outcomes differ across stages of development. This gap makes it difficult for coaches and practitioners to interpret test results correctly or to implement fair, developmentally appropriate training and selection strategies [18,19,20].

Therefore, the present study aimed to examine the influence of biological maturity status on anthropometric characteristics, body composition, and physical performance in elite youth male basketball players. Using a cross-sectional design, athletes were categorized as early, on-time, or late maturers according to their predicted PHV. Physical performance was evaluated through a multidimensional testing battery that included measures of body composition, lower-body power, linear sprint speed, and change-of-direction ability.

It was hypothesized that early maturers, reflecting more advanced biological development, would demonstrate greater stature, muscle mass, and superior performance across explosive, sprint, and agility measures compared with on-time and late maturers. By providing maturity-specific reference data, this study aims to help practitioners contextualize player performance, individualize training prescriptions, and support fair and developmentally appropriate talent identification within elite youth basketball systems.

## 2. Materials and Methods

### 2.1. Experimental Approach to the Problem

A cross-sectional study design was employed to examine the influence of biological maturity status on anthropometric and performance characteristics among elite youth male basketball players. Elite athletes in this study were defined based on their participation in the highest national youth basketball competition and a regular training volume of 4–6 sessions per week, including technical–tactical and strength-conditioning training. All participants completed a standardized testing battery assessing body composition, jumping ability, sprint performance, and agility. The study aimed to identify performance differences between early, on-time, and late-maturing athletes and to explore the role of biological maturation in talent identification and physical development.

Testing was conducted during the early competitive phase of the season, ensuring that players were in stable training conditions and free from injury. All assessments were carried out at least 48–72 h after the last competitive match or intense training session to minimize fatigue effects. Prior to testing, athletes performed a standardized 15 min warm-up consisting of 10 min of light jogging and dynamic mobility exercises, followed by progressive jumps and short sprints to ensure optimal readiness.

The testing battery included anthropometric assessments (standing height, sitting height, leg length, and body mass) and body composition analysis (body fat percentage and skeletal muscle mass). Performance tests comprised countermovement jump (CMJ) with and without arm swing, drop jump (DJ40), linear sprint tests over 5, 10, 15, and 20 m, and two agility tests: the *t*-test and lane agility drill. Each participant completed familiarization trials before testing, and all measurements were performed under identical indoor conditions on a standard hardwood court to ensure consistency.

Maturity status was estimated using the Mirwald equation [21], which predicts years from peak height velocity (PHV) based on anthropometric variables. Players were then categorized into three maturity groups: early, on-time, and late maturers. The classification enabled comparison of physical and performance characteristics across maturity levels, representing distinct stages of biological development within the adolescent growth period. All tests were administered by the same team of qualified evaluators experienced in basketball performance assessment.

### 2.2. Sample

A total of 140 elite youth male basketball players (age = 15.12 ± 0.78 years; height = 181.3 ± 7.6 cm; body mass = 71.6 ± 10.8 kg) voluntarily participated in this study. Participants were recruited using a purposive sampling approach from seven officially registered basketball academies competing at the highest national level in Bosnia and Herzegovina. These academies represent the main elite youth basketball development pathway in the country. The sample was not intended to represent elite youth basketball players internationally, but it is considered representative of elite youth male basketball players within Bosnia and Herzegovina. All eligible players present during testing agreed to participate, and no withdrawals occurred.

Biological maturity status was estimated using the noninvasive predictive equation developed by Mirwald et al. [21], which calculates the number of years from peak height velocity (PHV) as an indicator of biological age. The maturity offset was determined according to the following equation:

Maturity Offset = −9.236 + 0.0002708 × (Height × Sitting Height) − 0.001663 × (Age × Height) + 0.007216 × (Age × Sitting Height) + 0.02292 × Weight/Height

Based on the predicted years from PHV (YPHV), players were classified into three biological maturity groups: early maturers (more than 1.0 year after PHV), on-time maturers (within ±1.0 year of PHV), and late maturers (more than 1.0 year before PHV). This method has demonstrated high reliability (R^2^ = 0.91; SEE = 0.50 years) and is widely recognized for its practicality and accuracy in adolescent athlete research [16,19]. All players had at least five years of organized basketball experience and trained four to six times per week, including technical–tactical and strength-conditioning sessions (60–90 min each). Each team played approximately 20–25 official matches during a 35-week competitive season. Participants were free from injury for at least six months prior to testing and were instructed to maintain their usual hydration and nutritional habits during the testing period. Inclusion criteria required players to be registered members of elite youth programs, without recent musculoskeletal injuries, and to have completed all testing sessions. Those who did not complete the full protocol or showed signs of acute illness were excluded from the analysis.

Prior to data collection, players and their legal guardians were fully informed of the study’s aims, procedures, and potential risks. Written informed consent was obtained from all participants and their guardians. The study was approved by the Ethics Committee of the Faculty of Sport and Physical Education, University of Sarajevo (No: 101-678-1/24 dated 20 January 2024) and conducted in accordance with the principles of the Declaration of Helsinki.

### 2.3. Measurement Procedure

#### 2.3.1. Anthropometric Measurements and Maturity Status

Anthropometric assessments were performed in accordance with the standardized procedures [22,23]. All measurements were conducted in a temperature-controlled indoor facility with participants wearing light sports clothing (shorts and t-shirts) and without shoes. Standing height and sitting height were recorded without shoes to the nearest 0.1 cm using a digital stadiometer (InBody BSM 370; Biospace Co., Ltd., Seoul, Republic of Korea). For the measurement of standing height, players stood in an erect posture with feet together, arms relaxed, and the head positioned in the Frankfurt horizontal plane. Sitting height was obtained while participants sat upright on a 40 cm-high bench with knees together and head aligned horizontally. Leg length was calculated as the difference between standing and sitting height.

Body mass and body composition were assessed using a multi-frequency bioelectrical impedance analyzer (InBody 720, Biospace Co., Seoul, Republic of Korea). The device provided estimates of body fat percentage (BF%), fat-free mass, and skeletal muscle mass. Participants were instructed to avoid food and liquid intake for at least 3 h, refrain from caffeine and alcohol for 24 h, and avoid intense physical activity for at least 12 h prior to testing. During the assessment, subjects stood barefoot on the device’s electrodes with arms slightly abducted to ensure proper contact and measurement accuracy [22].

All anthropometric and body composition measurements were taken by the same experienced examiner to minimize inter-observer variability. Each measurement was repeated twice, and the mean value was recorded for further analysis.

#### 2.3.2. Linear Sprint Speed

Linear sprint performance was evaluated using 5 m, 10 m, 15 m, and 20 m sprint tests. Sprint times were recorded using a dual-beam electronic timing system (Witty, Microgate, Bolzano, Italy), which has demonstrated high test–retest reliability (ICC = 0.83–0.90; CV = 1.5–1.9%) [24], for short-distance sprint assessments in youth athletes. The photoelectric cells were positioned at a height of 0.9 m and aligned in pairs, separated by 1 m to ensure precise detection of passage.

Each athlete began from a standing start position, with the front foot placed 0.5 m behind the first timing gate to avoid triggering the sensors prematurely. Participants were instructed to accelerate maximally from the start and maintain full effort through the final gate. Each player completed three maximal sprint trials, with two minutes of passive recovery between attempts to minimize fatigue.

Prior to testing, all players performed a standardized warm-up consisting of dynamic stretching, progressive accelerations, and two submaximal sprint trials for familiarization. Sprint times were automatically recorded to the nearest 0.01 s, and the fastest trial at each distance was retained for further statistical analysis.

The chosen distances (5, 10, 15, and 20 m) represent distinct phases of acceleration and maximal sprinting relevant to basketball-specific movement demands. All sprint tests were conducted indoors on a standard hardwood court.

#### 2.3.3. Jumping Performance

Lower-body power was assessed using countermovement jump (CMJ) tests with and without arm swing, and a drop jump performed from a 40 cm height (DJ40). All jump tests were conducted indoors on a standard hardwood basketball surface to ensure consistent conditions and minimize the influence of external environmental factors. A photoelectric timing system (Optojump Next, Microgate, Bolzano, Italy) was used to measure flight time and calculate jump height. This system has been validated as a reliable and accurate tool for assessing vertical jump performance compared with force platforms (ICC = 0.98; CV = 2.7%) [25].

Prior to testing, participants performed three submaximal practice trials to familiarize themselves with the protocol. Each player then completed three maximal CMJ attempts, separated by 90 s of passive recovery. For the CMJ without arm swing, players were instructed to keep their hands placed firmly on their hips to eliminate the contribution of upper-body momentum. For the CMJ with free arm swing, players were allowed to use natural arm motion to maximize jump height. The starting position for each jump was upright, with the head in the neutral position, knees fully extended, and feet shoulder-width apart. On the tester’s command, players performed a rapid downward movement followed by a maximal upward jump, attempting to reach maximum vertical displacement.

The drop jump (DJ40) was performed from a 40 cm platform. Upon stepping off the platform, athletes were instructed to minimize ground contact time and immediately perform a maximal vertical rebound jump. Each player performed two valid DJ40 trials, and the best result was retained for analysis.

Trials were repeated if athletes exhibited excessive knee flexion upon landing, incomplete extension during take-off, or noticeable horizontal movement during the jump. The highest jump value for each test condition was recorded for statistical analysis.

#### 2.3.4. Agility

Change in direction (CoD) ability was evaluated using the *t*-test and Lane Agility test, both of which are widely recognized for assessing multidirectional speed, coordination, and movement efficiency in basketball players [26]. All tests were performed indoors on a hardwood court under standardized conditions, with athletes wearing their regular basketball shoes. Execution times were recorded using a dual-beam photoelectric timing system (Witty, Microgate, Bolzano, Italy), with precision to the nearest 0.01 s.

For the *t*-test, participants began from a standing start position 0.5 m behind the first timing gate. Upon the tester’s signal, they sprinted forward 10 m to touch the top of a central cone, shuffled laterally 5 m to the left to touch another cone, then shuffled 10 m to the right to touch a third cone, and finally returned 5 m left to the center cone before backpedaling 10 m to the start line [27]. Throughout the test, players were instructed to face forward at all times and avoid crossing their legs during lateral movement. Each athlete performed three maximal trials, separated by 2–3 min of passive recovery, and the fastest time was retained for analysis.

The Lane Agility test was used as a basketball-specific assessment of multidirectional quickness and defensive movement [28]. Cones were arranged to form the shape of a standard key (lane) on a basketball court. Players started with one foot behind the baseline cone, facing forward. On the command “Go”, they sprinted diagonally to the opposite front cone, shuffled laterally across the free-throw line, backpedaled to the baseline, and repeated the same sequence in the opposite direction. The goal was to complete the circuit as quickly as possible while maintaining proper defensive posture and balance. Each player completed two practice trials followed by two recorded trials, with 2 min recovery intervals. The best performance time was used for statistical analysis.

### 2.4. Statistical Analysis

Data were expressed as mean ± standard deviation (SD) for each maturity group (early, on-time, and late maturers). Prior to analysis, all variables were screened for normality and homogeneity of variances using the Shapiro–Wilk and Levene tests, respectively. To reduce potential bias caused by nonuniformity error, data were log-transformed before further analysis.

Differences between maturity groups were examined using one-way analysis of variance (ANOVA). Where significant main effects were detected, Bonferroni post hoc comparisons were applied to identify pairwise differences. The magnitude of differences between groups was assessed using partial eta squared (ηp^2^) as a measure of effect size, interpreted according to the following thresholds [29]: small (ηp^2^ < 0.06), moderate (0.06 ≤ ηp^2^ < 0.14), and large (ηp^2^ ≥ 0.14).

To complement traditional null-hypothesis testing, magnitude-based inferences (MBI) were also employed to evaluate the practical significance of differences between consecutive maturity groups. The smallest worthwhile difference (SWD) was defined as 0.2 × between-subject SD, consistent with Cohen’s d principle for standardized effect sizes. The likelihood that the observed effect exceeded the SWD was qualitatively interpreted as follows [30,31]: <0.5% almost certainly not; 0.5–5% very unlikely; 5–25% unlikely; 25–75% possibly; 75–95% likely; 95–99.5% very likely; and >99.5% almost certainly.

Standardized effect sizes (ESs) were interpreted using the following criteria [30]: trivial (<0.2), small (0.2–0.6), moderate (0.6–1.2), large (1.2–2.0), and very large (>2.0). When the 90% confidence interval overlapped both positive and negative SWD boundaries (ES ± 0.2), the magnitude of difference was described as unclear. All statistical analyses were performed using IBM SPSS Statistics (version 27.0; IBM Corp., Armonk, NY, USA), and the level of statistical significance was set at *p* < 0.05.

## 3. Results

Descriptive statistics and one-way ANOVA outcomes for all variables across maturity groups are presented in Table 1. Maturity status had a significant main effect on the majority of anthropometric and performance variables (*p* < 0.05). The overall model explained a substantial proportion of variance in physical characteristics (F = 88.57, *p* < 0.001), indicating clear maturational differences among players.

Early-maturing athletes were significantly older, taller, and heavier compared to both on-time and late maturers (*p* < 0.001). The most pronounced differences were observed for height (ηp^2^ = 0.667), body mass (ηp^2^ = 0.455), and sitting height (ηp^2^ = 0.321), reflecting the expected somatic growth advantages associated with advanced biological age. BMI and muscle mass also increased progressively with maturity status, while body fat percentage showed a small but consistent reduction among early maturers. The variability within groups (SD) decreased slightly with increasing maturity, suggesting greater morphological homogeneity in more physically developed athletes.

Jump performance improved in line with maturation level. Both CMJ and DJ40 showed significant group differences (*p* < 0.05), with early maturers exhibiting higher mean values (32.2 ± 5.4 cm and 31.7 ± 5.9 cm, respectively) than late maturers (28.5 ± 4.4 cm and 27.8 ± 4.5 cm). Effect sizes ranged from moderate to large (ηp^2^ = 0.051–0.087), indicating that vertical power output is sensitive to maturational development. On-time players displayed intermediate values, following the same upward trend.

Sprint performance revealed clear maturational effects across all measured distances. Significant differences were found for 5, 10, 15, and 20 m sprints (*p* < 0.01), with mean times progressively decreasing from late to early maturers. The largest effects were observed for longer sprint distances (ηp^2^ = 0.166), emphasizing the influence of biological maturity on both acceleration and maximal sprint phases. Post hoc comparisons confirmed that early maturers were faster than on-time and late groups across all sprint segments.

Change-of-direction performance also differed significantly between maturity groups. In both the *t*-test and Lane Agility test, early maturers demonstrated shorter completion times compared with late and on-time players (*p* < 0.05), with large effect sizes (ηp^2^ = 0.100–0.124). These findings indicate a progressive improvement in movement efficiency and agility with advancing maturity.

Bonferroni post hoc analysis revealed that late maturers consistently scored lower than both on-time and early maturers across nearly all measured parameters. On-time players also differed from early maturers in several anthropometric and performance indicators, particularly in body dimensions and sprint times.

MBI results are presented in Table 2. Pairwise comparisons between consecutive maturity groups demonstrated clear and practically meaningful differences across most variables. Very large effects and “almost certain” differences were found between early and late maturers for age, height, sitting height, leg length, and muscle mass, indicating substantial advantages among early maturers. Moderate-to-large differences were observed for jumping and sprint performance, while small but consistent effects were noted for agility measures. Comparisons between on-time and early maturers generally showed smaller and less consistent differences, categorized as “possibly” or “likely” in magnitude terms.

## 4. Discussion

The present study examined the influence of biological maturity status on anthropometric, body composition, and physical performance characteristics in elite youth male basketball players. The results demonstrated clear differences between maturity groups, confirming that biological maturity has a substantial effect on both morphological and functional attributes during adolescence. Players who matured earlier exhibited greater height, body mass, and skeletal muscle mass, as well as superior performance in jumping, sprinting, and agility tasks compared with on-time and later-maturing peers. These findings align with previous research [3,16,19,32] and reinforce the importance of considering maturation when evaluating physical performance and designing developmental strategies in youth basketball.

In addition to the traditional ANOVA outcomes, MBI results presented in Table 2 further emphasized the practical significance of these differences. Most comparisons between early and late maturers were classified as “likely” or “almost certain”, particularly for body dimensions and muscle mass, indicating that these effects are not only statistically significant but also meaningful in real sporting contexts. Moderate-to-large standardized effects were also evident for jumping and sprint performance, while agility measures showed smaller yet consistently positive tendencies toward earlier maturity.

The observed anthropometric differences across maturity groups reflect normal biological processes associated with puberty and somatic growth. Early maturers were significantly taller, heavier, and more muscular, consistent with the well-documented acceleration in lean tissue growth and endocrine activity that accompanies advanced biological maturation [9,33]. Similar maturational trends have been reported in both basketball and football populations [3,18,19], suggesting that these advantages are a universal feature of adolescent athletic development. The MBI interpretation complements these statistical outcomes by demonstrating that the probability of such maturational effects being practically relevant was high, thereby reinforcing the robustness of the observed trends.

Performance measures related to lower-body power and explosive ability followed a similar maturational pattern. Both the CMJ and DJ40 showed higher values among early maturers, with on-time athletes positioned between the two extremes. This is consistent with previous studies indicating that biological maturation enhances muscle strength, tendon stiffness, and neuromuscular coordination, thereby increasing the capacity for explosive power production [18,32]. In this regard, Table 2 shows that the likelihood of meaningful differences in jump performance between maturity groups ranged from “likely” to “very likely,” suggesting that even moderate statistical effects carry practical implications for player monitoring and training.

Similarly, sprint and agility performances improved progressively with increasing maturity. Early maturers displayed faster times in the 5–20 m sprints and greater efficiency in both agility tests. These differences likely reflect longer stride length, greater muscle force, and improved neuromuscular coordination, all of which are influenced by hormonal and structural maturation [3,34,35,36,37,38,39,40]. Consistent with the MBI findings, the probabilities for meaningful differences in sprint performance were rated as “likely” to “very likely,” underlining the influence of maturation even in short-duration, high-velocity tasks. Although these results are consistent with existing literature, they also carry an important caution. As emphasized by Cumming et al. [4] and Towlson et al. [14], maturational differences can distort the talent identification process, leading to an overrepresentation of early developers in elite youth programs. This phenomenon—often referred to as maturity selection bias—can result in the premature exclusion of late-maturing players who may ultimately possess equal or even superior long-term potential once their physical development catches up.

The maturity-related differences observed in the present study are consistent with previous findings in youth basketball and other team sports such as football and volleyball [3,14,18]. Earlier-maturing athletes have been shown to display advantages in strength-, power-, and speed-related tasks during adolescence, primarily reflecting advanced biological development rather than training effects alone [3,4,19]. Similar maturity-associated patterns have been reported across team sports, supporting the relevance of maturity-informed approaches when interpreting physical performance during adolescent development [4,14].

From a practical standpoint, the findings of this study emphasize the need for maturity-informed practices in youth basketball. Integrating approaches such as bio-banding or maturity-offset classification can help reduce the influence of temporary biological advantages by grouping athletes according to their developmental stage rather than chronological age [4,36,40]. This allows for more equitable training and competition environments and encourages the development of technical and tactical skills among players of all maturity levels, without increasing anxiety and competitive stress [41]. Coaches should also consider biological maturity when planning and monitoring training loads, particularly in strength and plyometric work, ensuring that younger biological athletes are not overexposed to high-intensity or high-volume programs before they are physiologically ready [35,37,38,39]. It should be emphasized that, due to the cross-sectional design, the present findings reflect associations between biological maturity and physical performance rather than predictive or causal relationships. Consequently, implications for talent identification should be interpreted with caution, and longitudinal follow-up studies are required to determine how maturity-related differences influence long-term player development and selection outcomes.

While early maturers may currently demonstrate superior physical performance, late maturers should not be viewed as disadvantaged in the long term. Several longitudinal studies [3,4,14] have shown that late-maturing athletes often display higher technical adaptability, coordination, and resilience, which may contribute to sustained success at the senior level. Recognizing and supporting this diversity is essential to maintaining broad and inclusive talent pathways. The current results therefore support a shift from outcome-based evaluation toward developmentally sensitive monitoring that values growth, learning, and progression across the entire spectrum of maturity.

The present study has several limitations. The cross-sectional design does not allow causal interpretations of the relationship between biological maturity and physical performance. In addition, the sample was drawn from a single country, which may limit the generalizability of the findings to other youth basketball systems. Biological maturity was estimated using the Mirwald et al. [21] predictive equation, which, although widely used and practical, may present limitations when applied to highly trained youth and sport-specific populations [8]. Finally, the study focused on anthropometric and physical performance variables and did not include technical or tactical performance measures, which should be considered in future research.

## 5. Conclusions

The present study demonstrates that biological maturity status is a decisive factor in shaping the anthropometric and performance characteristics of elite youth male basketball players. Early-maturing athletes exhibited significantly greater height, muscle mass, and superior results in jumping, sprinting, and agility tasks compared with their on-time and late-maturing peers. These findings reflect the physiological acceleration associated with earlier maturation rather than differences in training exposure or motivation.

From an applied perspective, the integration of MBI revealed that these maturity-related advantages are not only statistically significant but also practically meaningful, particularly for variables related to power and speed. Such insights emphasize the need for coaches and practitioners to move beyond purely chronological age groupings and consider biological age when designing talent identification and development programs. To promote long-term athlete development, implementing maturity-informed approaches such as bio-banding, individualized strength and conditioning progressions, and continual monitoring of growth and performance is essential. These strategies can help minimize maturity-related bias, support fair player evaluation, and ensure that late-developing athletes remain engaged within the performance pathway. Ultimately, this study provides maturity-specific reference data that can assist practitioners in interpreting physical testing results and optimizing developmental training models in elite youth basketball. This study addressed the research question by demonstrating consistent associations between biological maturity status and anthropometric and physical performance characteristics in elite youth male basketball players. The use of magnitude-based inference alongside conventional statistical analysis represents a novel aspect of this study, facilitating interpretation of the practical relevance of maturity-related differences.

## Figures and Tables

**Table 1:** Anthropometric and performance characteristics across maturity groups (Late, On-time, Early): ANOVA results.

Variables	Late (n = 43)		On-Time (n = 51)		Early (n = 46)		ANOVA		
	Mean	SD	Mean	SD	Mean	SD	η_p_^2^	F	Bonferroni (*p* ≤ 0.01)
Age (year)	14.06	0.63	15.28	0.79	15.91	0.54	0.564	88.572	Late < On-time; Late < Early; On-time < Early
Height (cm)	172.43	6.62	182.12	3.91	190.27	4.56	0.667	137.098	Late < On-time; Late < Early; On-time < Early
Sitting height (cm)	79.32	3.04	83.77	1.80	87.52	2.10	0.321	32.406	Late < On-time; Late < Early; On-time < Early
Leg length (cm)	93.11	3.57	98.34	2.11	102.75	2.46	0.072	5.343	Late < On-time; Late < Early; On-time < Early
Body mass (kg)	61.18	11.79	74.23	11.22	79.33	9.76	0.455	57.292	Late < On-time; Late < Early; On-time < Early
BMI (kg/m^2^)	20.45	3.01	22.35	3.11	21.91	2.53	0.667	137.098	Late < On-time; Late < Early
BF% (%)	13.43	5.98	14.35	6.21	11.82	4.78	0.667	137.098	Late < On-time
MM (kg)	29.24	5.11	35.48	4.12	39.51	4.55	0.034	2.410	Late < On-time; Late < Early; On-time < Early
CMJ free (cm)	36.30	5.42	37.28	6.21	39.85	7.41	0.087	6.493	Late < Early
CMJ (cm)	28.50	4.39	30.06	4.78	32.21	5.40	0.051	3.685	—
DJ40 (cm)	27.76	4.54	29.91	5.04	31.74	5.88	0.087	6.530	Late < Early
5 m (s)	1.18	0.11	1.15	0.09	1.12	0.09	0.051	3.656	Late < Early
10 m (s)	2.03	0.19	1.95	0.13	1.91	0.12	0.105	7.999	Late < On-time; On-time > Early
15 m (s)	2.72	0.20	2.61	0.17	2.54	0.16	0.154	12.505	Late < On-time; Late < Early
20 m (s)	3.45	0.26	3.31	0.20	3.20	0.20	0.166	13.642	Late < On-time; Late < Early
*t*-test (s)	11.55	0.85	11.09	0.76	10.78	0.88	0.124	9.697	Late < Early
Lane agility (s)	13.50	0.91	13.13	0.91	12.75	0.91	0.100	7.579	Late < Early

BMI—Body mass index; BF%—Body fat percentage; MM—Muscle mass; CMJ—Countermovement jump; DJ40—Drop jump 40 cm.

**Table 2:** Effect size and magnitude-based inferences between maturity groups.

Variables	Late vs. Early	Late vs. On-Time	On-Time vs. Early
Age (year)	Very large/Almost certain (1.73 ± 0.12)	Large/Very likely (0.89 ± 0.10)	Very large/Almost certain (3.15 ± 0.16)
Height (cm)	Very large/Almost certain (3.14 ± 2.02)	Large/Very likely (1.78 ± 1.91)	Large/Very likely (1.92 ± 1.44)
Sitting height (cm)	Very large/Almost certain (3.14 ± 0.93)	Large/Very likely (1.78 ± 0.88)	Large/Very likely (1.92 ± 0.66)
Leg length (cm)	Very large/Almost certain (3.14 ± 1.09)	Large/Very likely (1.78 ± 1.03)	Large/Very likely (1.92 ± 0.78)
Body mass (kg)	Large/Very likely (1.68 ± 3.83)	Moderate/Likely (1.13 ± 3.97)	Small/Possibly (0.49 ± 3.54)
BMI (kg/m^2^)	Small/Possibly (0.53 ± 0.98)	Moderate/Likely (0.62 ± 1.05)	Trivial (−0.16 ± 0.95)
BF% (%)	Small/Possibly (−0.30 ± 0.22)	Trivial/Unclear (0.15 ± 0.24)	Small/Possibly (−0.46 ± 0.23)
MM (kg)	Very large/Almost certain (2.12 ± 1.71)	Large/Very likely (1.35 ± 1.61)	Moderate/Likely (0.93 ± 1.47)
CMJ free (cm)	Small/Possibly (0.55 ± 2.28)	Trivial (0.17 ± 1.99)	Small/Possibly (0.38 ± 2.32)
CMJ (cm)	Moderate/Likely (0.75 ± 1.73)	Small/Possibly (0.34 ± 1.57)	Small/Possibly (0.42 ± 1.73)
DJ40 (cm)	Moderate/Likely (0.76 ± 1.84)	Small/Possibly (0.45 ± 1.64)	Small/Possibly (0.33 ± 1.86)
5 m (s)	Small/Possibly (−0.56 ± 0.03)	Small/Possibly (−0.28 ± 0.03)	Small/Possibly (−0.3 ± 0.03)
10 m (s)	Moderate/Likely (−0.79 ± 0.06)	Small/Possibly (−0.47 ± 0.06)	Small/Possibly (−0.38 ± 0.04)
15 m (s)	Moderate/Likely (−1.02 ± 0.06)	Moderate/Likely (−0.62 ± 0.06)	Small/Possibly (−0.43 ± 0.05)
20 m (s)	Moderate/Likely (−1.05 ± 0.08)	Small/Possibly (−0.58 ± 0.08)	Small/Possibly (−0.55 ± 0.07)
*t*-test (s)	Moderate/Likely (−0.89 ± 0.3)	Small/Possibly (−0.57 ± 0.28)	Small/Possibly (−0.38 ± 0.28)
Lane agility (s)	Moderate/Likely (−0.83 ± 0.32)	Small/Possibly (−0.41 ± 0.31)	Small/Possibly (−0.42 ± 0.31)

MBI qualitative probabilities were interpreted as follows: <0.5% = almost certainly not; 0.5–5% = very unlikely; 5–25% = unlikely; 25–75% = possibly; 75–95% = likely; 95–99.5% = very likely; >99.5% = almost certain; ±—90% confidence intervals; BMI—Body mass index; BF%—Body fat percentage; MM—Muscle mass; CMJ—Countermovement jump; DJ40—Drop jump 40 cm.

## Data Availability

The original contributions presented in this study are included in the article. Further inquiries can be directed to the corresponding author(s).

## Abbreviations

The following abbreviations are used in this manuscript:

PHV	Peak height velocity
CMJ	Countermovement jump
DJ40	Drop jump from 40 cm
MBI	Magnitude-based inference
YPHV	Years from PHV
BF%	Body fat percentage
CoD	Change in direction
SD	Standard deviation
ηp^2^	Partial eta square
SWD	Smallest worthwhile difference
ES	Effect sizes
